# An explainable machine learning model based on multidimensional task-state electroencephalography for identifying attention-deficit/hyperactivity disorder comorbid developmental dyslexia

**DOI:** 10.3389/fnhum.2026.1976311

**Published:** 2026-10-02

**Authors:** Keduo Huang, Pengxiang Zuo, Xuemei Luo, Chun Ji, Fang Yang, Yuhao Huang, Xiaole Wang

**Affiliations:** 1 Department of Nursing, School of Medicine, Shihezi University, Shihezi, China; 2 Department of Child Health Care, The Second Xiangya Hospital of Central South University, Changsha, China

**Keywords:** attention deficit hyperactivity disorder, comorbidity, developmental dyslexia, electroencephalography, machine learning, clinical identification

## Abstract

**Background:**

Attention-deficit/hyperactivity disorder (ADHD) and developmental dyslexia (DD) are highly comorbid, yet objective differentiation from individual disorders remains challenging due to the lack of neurophysiological biomarkers. This study aimed to develop an interpretable machine learning model leveraging multidimensional task-state EEG features to distinguish ADHD–DD comorbidity from ADHD and typical development (TD).

**Methods:**

Ninety children (27 ADHD-DD, 40 ADHD, 23 TD) underwent EEG during a VSWM task. Five feature categories were extracted: event-related potentials, time-frequency characteristics, functional connectivity, graph-theoretic metrics, and nonlinear dynamics. Three feature-processing strategies and nine classifiers were compared within a nested cross-validation framework. Feature contribution was quantified by coefficient back-projection of the optimal PCA-LR mode. Sensitivity and permutation analyses were performed.

**Results:**

The PCA-LR model achieved a balanced accuracy of 80.5%, a Macro-F1 of 0.799, and a Macro-AUC of 0.912. Class-wise AUCs were 0.905 for ADHD-DD, 0.904 for ADHD, and 0.927 for TD. Left temporal α-band functional connectivity was a shared high-contribution feature in both disorder groups. ADHD-DD was distinguished by widely distributed cross-regional connectivity spanning θ and α bands and by P200 amplitude. ADHD was characterized primarily by α-band network metrics and α-ERD, whereas TD relied mainly on θ and α bands functional connectivity and graph-theoretic metrics.

**Conclusion:**

Our findings suggest that multidimensional task-state EEG features may help differentiate children with ADHD-DD comorbidity from those with ADHD or typical development. They also indicate that machine-learning approaches may be useful for identifying neurodevelopmental comorbid conditions.

## 1 Introduction

Attention deficit hyperactivity disorder (ADHD) and developmental dyslexia (DD) are common neurodevelopmental disorders in childhood. The two disorders share a high comorbidity rate of 25–40% (Slaby et al., 2023). Compared to children with a single disorder, those with comorbid conditions exhibit more severe neurocognitive deficits (Fernandez-Andres et al., 2021; Slaby et al., 2023), and are at greater risk for interpersonal difficulties, behavioral problems, psychological distress, and school dropout (Hongyao et al., 2023). Current clinical diagnosis relies primarily on the descriptive criteria of the Diagnostic and Statistical Manual of Mental Disorders, Fifth Edition (DSM-5) (Organization, A.P, 2013) and behavioral rating scales, yet lacks objective biomarkers capable of identifying comorbid features. Furthermore, children with ADHD–DD comorbidity face both attention problems and reading skill deficits, with symptoms and cognitive impairments that substantially overlap with those seen in either disorder alone (Zhang et al., 2025). Consequently, these individuals are frequently misdiagnosed as having a single disorder in clinical practice. Objective biomarkers that can reliably distinguish ADHD–DD comorbidity from individual disorders are urgently needed to improve early identification.

Visuospatial working memory (VWM) is primarily responsible for the temporary storage and manipulation of visuospatial information, underpinned by the coordinated activity of distributed brain regions. Research indicates that VWM is closely related to spatial navigation, object recognition, visual attention, and reading (Xu et al., 2020). VWM deficits have been documented in both ADHD and DD (Poon et al., 2024; Yang et al., 2024), representing one of the core overlapping cognitive impairments between the two disorders. However, whether the neural processing patterns during VWM tasks in children with comorbid ADHD–DD differ from those with either single disorder remains unclear. Electroencephalography (EEG) has been widely used in research on higher-order cognitive functions such as attention, emotion, and memory, owing to its safety, portability, high temporal resolution, and high sensitivity to dynamic changes in neural signals (Gkintoni et al., 2025; Zhang et al., 2023). Unlike resting-state EEG, which primarily reflects spontaneous neural activity, task-state EEG can capture task-evoked neural responses associated with specific cognitive functions, thereby unmasking latent compensatory or deficient neural processes. Therefore, investigating neuroelectrophysiological differences among children with ADHD–DD comorbidity, single-disorder ADHD, and typical development through VWM task-state EEG may provide preliminary electrophysiological evidence relevant to the comorbid condition.

Previous studies have identified detectable abnormal patterns in EEG features of patients with ADHD and DD, such as reduced frontal θ-ERS and attenuated P300 amplitude (Santoni et al., 2024; Zhao et al., 2025). However, these findings were largely derived from univariate statistical comparisons, which cannot capture complex interactions among multidimensional features or their predictive utility at the individual level. The development of machine learning (ML) techniques has provided an effective means to integrate EEG signals and achieve individualized classification and prediction. Although EEG-based machine learning models have shown promise in distinguishing ADHD or DD from healthy controls (Ghasemi et al., 2022; Jan et al., 2026), existing approaches still have several limitations. First, feature fusion remains insufficient, as most studies have employed single features without integrating multi-level signals. Second, existing research has predominantly focused on binary classification against healthy populations; the neural signatures of ADHD–DD comorbidity remain largely unexplored. To address these challenges, the present study integrated multidimensional EEG features encompassing temporal, spectral, functional connectivity, and nonlinear measures, and constructed a machine learning classification model to distinguish ADHD comorbid with DD, ADHD alone, and typically developing children. Furthermore, by introducing SHapley Additive exPlanations (SHAP) to quantify each feature’s contribution to model predictions, this study aims to provide an interpretable framework for objective identification of ADHD-DD comorbidity.

## 2 Materials and methods

### 2.1 Participants

This study recruited participants with ADHD comorbid with DD (ADHD–DD group) and participants with ADHD alone (ADHD group) from the pediatric health care/psychiatry outpatient clinic of a hospital. Age-matched healthy volunteers were concurrently recruited as the typically developing control group (TD group).

#### 2.1.1 Inclusion criteria for the ADHD group

(1) Diagnosed by a clinician as meeting the DSM-V diagnostic criteria for ADHD; (2) a mean score ≥ 1.6 on the Swanson Nolan and Pelham, Version IV (SNAP-IV), or mean score between 1.1–1.5 on any subscale with at least six items scoring 2 or 3 points (Swanson et al., 2001); (3) a mean score ≥ 2 on a specific dimension of the Conners’ Parent Symptom Questionnaire (PSQ) (Fumeaux et al., 2021); (4) a score ≥ 65 on the Pupil Rating Scale Revised Screening for Learning Disabilities, (PRS) (Jin et al., 1998); (5) a T-score ≤ 70 on the Dyslexia Checklist for Chinese Children (DCCC) (Wu et al., 2006); (6) a mean score < 2.45 on the Mandarin dyslexia screening behavior checklist for primary school students (Fan et al., 2024); (7) a percentile rank ≥ 75 on the Combined Raven’s Test (CRT) (Wang et al., 2007); (8) enrollment in grades 2–6 of primary school.

#### 2.1.2 Inclusion criteria for the ADHD–DD group

(1) Diagnosed by a clinician as concurrently meeting the DSM-5 diagnostic criteria for both ADHD and DD; (2) Met inclusion criteria (1)–(3) and (7)–(8) for the ADHD group; (3) a score < 65 on the PRS; (4) a T-score > 70 on the DCCC; (5) a mean score ≥ 2.45 on the Mandarin dyslexia screening behavior checklist for primary school students.

#### 2.1.3 Inclusion criteria for the TD group

(1) Clinical diagnosis excluding ADHD and DD according to the same criteria; (2) enrollment in grades 2–6 of primary school.

#### 2.1.4 Exclusion criteria for all three groups

(1) Left-handedness; (2) past or current diagnosis of visual/hearing impairment; (3) past or current diagnosis of schizophrenia, tic disorder, autism spectrum disorder, or other related conditions; (4) have taken any ADHD-related medications (such as methylphenidate, atomoxetine, etc.) within the past 24 h (Robertson, 2019).

This study was carried out in accordance with the recommendations of the Declaration of Helsinki and was approved by the Ethics Committee of the Second Xiangya Hospital of Central South University. All participants voluntarily enrolled in the study, and written informed consent was obtained from their parents or legal guardians.

### 2.2 EEG experiment design and signal acquisition

#### 2.2.1 Task paradigm

In this study, the visuospatial working memory task was implemented using a classic paradigm, change detection (Lin and Oberauer, 2022), programmed with E-Prime 3.0. The task consisted of two blocks: color judgment and location judgment, each containing 60 trials. Participants were instructed to memorize the colors or locations of visual stimuli and judge whether a subsequent target matched the initial display (Figure 1). In the present study, EEG data from the color and location blocks were combined to examine visuospatial working memory as a whole.

**Figure 1:**
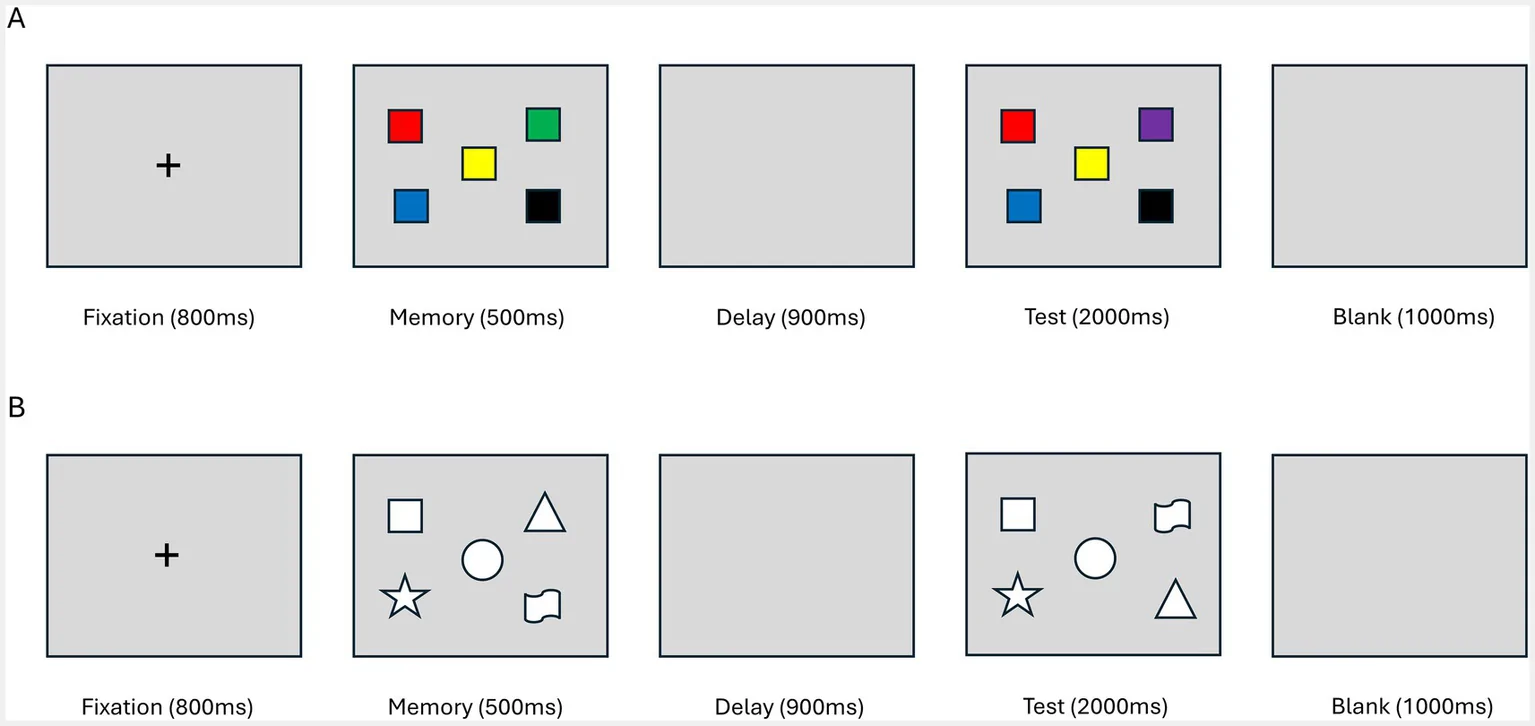
Visuospatial working memory task content. **(A)** Color change detection. **(B)** Location change detection.

#### 2.2.2 EEG data recording and preprocessing

EEG data were recorded using a 64-channel EGI system, with electrodes positioned according to the international 10–20 system. The impedance of each electrode was lowered to below 5 kΩ, and the sampling rate was set at 1000 Hz. Participants underwent a practice session to ensure adequate task comprehension and data quality prior to formal recording. After each block, a 5-min break was given.

Preprocessing was performed using MATLAB R2023a and the EEGLAB toolbox. The specific steps were as follows: (1) Re-referencing to the whole-brain average, applying a 0.5–35 Hz bandpass filter, and down-sampling to 500 Hz; (2) Segmenting the data from 500 ms pre-stimulus to 4,100 ms post-stimulus according to stimulus markers; (3) Baseline correction; (4) Removing and interpolating bad channels; (5) Rejecting bad epochs; (6) Applying independent component analysis (ICA) to remove electrooculogram, electrocardiogram, and electromyogram artifacts; (7) Final data screening with artifact removal based on extreme value criteria.

### 2.3 EEG features analysis

#### 2.3.1 Event-related potentials

For the event-related potential (ERP) analysis, epochs were extracted from 200 ms pre-stimulus to 1,000 ms post-stimulus, with the 200 ms pre-stimulus interval serving as the baseline for correction. The EEG signals were then averaged across trials for each participant to obtain ERP waveforms. Based on the grand-averaged waveforms and previous literature (Lenartowicz et al., 2016; McLoughlin et al., 2010; Papp et al., 2020; Senderecka et al., 2012), four ERP components were identified in this study: P1 (time window: 80–140 ms), N2 (time window: 180–300 ms), P2 (time window: 180–280 ms), and P3 (time window: 280–400 ms). The mean amplitude (μV) and peak latency (ms) within the specified time window were extracted from the electrodes of interest for each component and used for subsequent statistical analyses.

#### 2.3.2 Time-frequency analysis

Short-time Fourier transform (STFT) was applied to the preprocessed EEG data for time-frequency decomposition with a window length of 400 ms and a step size of 200 ms. The time-frequency power values were baseline-corrected using the pre-stimulus interval of 400–200 ms (Li et al., 2024) and converted into event-related desynchronization/synchronization (ERD/ERS) metrics. Based on the grand-averaged time-frequency map, the mean ERS value within the θ band (4–8 Hz, 200–300 ms post-stimulus) and the mean ERD value within the α band (8–12 Hz, 400–800 ms post-stimulus) were extracted for subsequent statistical analysis.

#### 2.3.3 Construction of brain functional networks

The preprocessed EEG data were decomposed in the time-frequency domain using the short-time Fourier transform (STFT; window length 0.4 s, step size 0.2 s), and instantaneous phase information was extracted from the complex time-frequency coefficients for each channel within the θ band (4–8 Hz, 200–300 ms post-stimulus) and the α band (8–12 Hz, 400–800 ms post-stimulus). Based on the phase series, the weighted phase-lag index (wPLI) between each pair of channels was computed at each time-frequency point to eliminate spurious zero-phase synchronization caused by volume conduction (Vinck et al., 2011). The wPLI ranges from 0 to 1, with values closer to 1 indicating stronger phase synchronization between the two channels.

The wPLI was calculated as follows:


$$\text{WPLI}=\frac{|\langle J(X)\rangle |}{\langle |J(X)|\rangle }=\frac{|\langle |J(X)|\cdot \mathrm{sign}(J(X))\rangle |}{\langle |J(X)|\rangle }$$

Where J(X) denotes the imaginary part of the cross-spectrum between signals x(t) and y(t) , reflecting the phase difference between the two signals.

Graph theoretical analysis was performed using the gretna toolbox (Matlab platform). The functional connectivity matrices were binarized using a sparsity threshold method. Consistent with previous studies, the sparsity threshold ranged from 0.07 to 0.40 with a step size of 0.01 (i.e., 7–40%, step 1%) (Zhang et al., 2011). Binary matrices were constructed to calculate three nodal properties: nodal efficiency (NE), betweenness centrality (BC), and degree centrality (DC). To minimize the bias associated with selecting a single threshold, the area under the curve (AUC) for each metric across the sparsity range was computed, and subsequent statistical analyses were performed on the AUC values.

#### 2.3.4 Nonlinear feature analysis

The analysis was conducted using a sliding window approach with a window length of 0.5 s and a step size of 0.05 s. Following epoch-wise averaging and baseline correction, the mean values within the post-stimulus interval of 0.3–0.5 s were extracted as the representative value for each channel (Talebi et al., 2012). The specific indices included: approximate entropy (ApEn; embedding dimension *m* = 2, tolerance *r* = 0.2 × SD), sample entropy (SaEn; *m* = 2, *r* = 0.2 × SD), permutation entropy (PeEn; *m* = 4, delay *τ* = 1), and permutation Lempel-Ziv complexity (PLZC; *m* = 4, *τ* = 1).

### 2.4 ML model construction and evaluation

All machine-learning models were implemented using the scikit-learn toolkit in the Python environment. Stratified 10-fold cross-validation was adopted as the outer loop to evaluate model generalization performance, while stratified 5-fold cross-validation served as the inner loop to conduct feature selection, classifier selection and hyperparameter optimization within each outer-loop training fold. Grid search was applied in the inner loop for hyperparameter tuning. A class-weight balancing strategy was introduced during model building to mitigate classification bias caused by imbalanced sample sizes. The outer-loop test folds were excluded from all feature-selection and parameter-tuning procedures to prevent data leakage. As reported by Varma and Simon, 2006, in which model selection is handled by the inner loop and generalization performance is assessed by the outer loop, can reduce optimistic-performance bias and yield more reliable estimates of generalization error for small-sample scenarios where a separate held-out test set is unavailable. Three feature-processing strategies were utilized: principal component analysis (PCA), least absolute shrinkage and selection operator (LASSO), and recursive feature elimination (RFE). PCA was used for linear dimensionality reduction; Lasso performed sparse feature selection via L1 regularization; RFE obtained the optimal feature subset by iteratively removing features with the lowest importance. Although whole-sample analysis of variance (ANOVA) was initially used as a noise-reduction step to define the candidate feature pool from the high-dimensional raw feature space, all actual feature-selection procedures (PCA, LASSO, RFE) were strictly confined to the inner-loop training folds. The number of retained principal components was optimized via the inner-loop cross-validation grid search, with candidate cumulative variance thresholds of 0.80, 0.90, and 0.95. Nine classifiers were included: linear discriminant analysis (LDA), logistic regression (LR), support vector machine (SVM), random forest (RF), AdaBoost, k-nearest neighbors (KNN), XGBoost, naive Bayes (NB), and decision tree (DT).

Model performance was evaluated using multiple metrics, including Macro-AUC, Macro-F1, specificity, precision, and balanced accuracy for model comparison, as well as class-level sensitivity, specificity, precision, F1, and one-vs-rest (OvR) AUC for the optimal model. Calibration was assessed via Brier score and calibration curves.

### 2.5 Feature importance interpretation

In the present study, coefficient back-projection was used to map weights from the principal-component space back onto the original feature space to enable model interpretability analysis.

This computation was strictly restricted to the inner-loop training folds of nested cross-validation. Within each fold, PCA was first fitted to obtain the loading matrix, and the three-class LR model was trained to yield class-specific coefficient vector $\beta_{k}$ . Weight back-projection was implemented via matrix multiplication between the transpose of the loading matrix and the coefficient vector. The mean of absolute values of weights output across all inner-loop folds was taken as the final discriminative contribution of each original EEG feature. This procedure reduced random fluctuations caused by a single data split and avoided leveraging test-set information so as to prevent data leakage.


$$W_{\text{original},k}=|P^{T}\beta_{k}|$$

Where P denotes the PCA loading matrix; $\beta_{k}$ represents the LR coefficients for the ***𝑘***-th class; $W_{\text{original},k}$ indicates the contribution weight of the original feature to the ***𝑘***-th class. A larger absolute weight value corresponds to higher discriminative contribution of the feature.

### 2.6 Robustness analysis

#### 2.6.1 Sensitivity analysis

To evaluate the robustness of the optimal LR model under different conditions, sensitivity analyses were performed from three dimensions. First, participants were stratified into female and male subgroups according to sex. Balanced accuracy, Macro-F1 and Macro-AUC were calculated for each subgroup based on the out-of-fold (OOF) prediction results from the outer-loop 10-fold cross-validation of the primary model. Second, sex was incorporated as a covariate into the LR model, which was retrained and evaluated under the identical nested cross-validation framework to compare model performance with and without the sex covariate. Finally, to assess the incremental value of EEG features relative to behavioral metrics, a behavior-only model (using only behavioral accuracy as input) and a combined EEG-plus-behavior model were constructed separately. All sensitivity analyses adopted the identical outer-loop 10-fold split as the primary model. All data-driven preprocessing and feature-selection procedures were strictly confined to training folds.

#### 2.6.2 Permutation test

Permutation tests were conducted to examine whether model performance was significantly above chance level. Stratified random permutation was applied to shuffle training-set labels for 100 times while preserving the sample proportion of each class. After each permutation, the full pipeline of model training and evaluation, including feature selection, hyperparameter optimization and outer-loop test-set prediction, was rerun under the same nested cross-validation framework. Accuracy, Macro-F1 and Macro-AUC on the outer-loop test set were recorded for each permutation. The empirical *p*-value was calculated by counting how many permutation runs yielded performance equal to or better than the true observed performance, with one added to both the numerator and the total number of permutations to avoid zero-valued *p*-values. All permutation procedures retained the same random seed and outer-loop data-splitting strategy as the primary model.

### 2.7 Statistical analysis

Continuous data conforming to a normal distribution were expressed as mean ± standard deviation ( $\bar{x}\pm s$ ), and comparisons among groups were performed using one-way ANOVA. Non-normally distributed data were expressed as median (P25, P75), and comparisons among groups were performed using Kruskal-Wallis *H* test. Categorical data were compared among groups using the chi-square ( $\chi^{2}$ ) test. Group differences in EEG features were assessed using one-way ANOVA. For group-level comparisons, the Benjamini-Hochberg procedure was used to control the false discovery rate (FDR) across all features. Post-hoc pairwise comparisons were corrected for multiple comparisons using the Bonferroni method. All statistical tests were two-tailed, and the significance level was set at *α* = 0.05. The 95% confidence intervals for all performance metrics were estimated using bootstrap methods (1,000 iterations).

## 3 Results

### 3.1 Participants

A total of 90 children were enrolled in this study, comprising 27 in the ADHD–DD group, 40 in the ADHD group, and 23 in the TD group. No significant differences were observed among the three groups in age, grade, or intelligence quotient (all *p* > 0.05). However, a significant difference was found in sex distribution across the groups (*χ*^2^ = 12.40, *p* = 0.002). VWM task accuracy showed significant group differences (*F* = 16.34, *p* < 0.001), with both ADHD–DD and ADHD groups performing worse than TD (both *p* < 0.05). No significant differences were detected in reaction time among the three groups (*F* = 0.272, *p* = 0.762).

### 3.2 EEG characteristics

To assess the group-level discriminability of multidimensional EEG features, one-way ANOVA was performed on ERP, time-frequency, functional connectivity, graph theory, and nonlinear measures. The results revealed significant group differences in ERP components (P100 latency, N200/P200/P300 amplitude), time-frequency features, functional connectivity, graph theory metrics (nodal efficiency, betweenness centrality, degree centrality), and nonlinear measures (ApEn, SaEn, PeEn, PLZC) (all *p* < 0.05). After Benjamini-Hochberg FDR correction, all 67 features remained statistically significant (all *q* ≤ 0.05, maximum *q* = 0.050). Detailed statistical results are provided in Supplementary Tables S1–S7.

### 3.3 Feature selection and model comparison

To compare classification performance across different models and identify the optimal model for discriminating children with ADHD-DD comorbidity, children with ADHD-only, and TD children, this study evaluated the classification performance of combinations of three feature-processing approaches (PCA, RFE, LASSO) and nine classifiers (LR, LDA, SVM, RF, AdaBoost, XGBoost, KNN, NB, DT) within a nested cross-validation framework. Macro-F1 was adopted as the criterion for inner-loop model selection. According to the averaged performance metrics from the outer-loop 10-fold test sets, PCA yielded the best overall performance among the three feature-processing methods, and LR achieved the optimal averaged performance in 9 out of 10 folds. The LR model with PCA dimensionality reduction was therefore designated as the final optimal model, which served as the basis for subsequent interpretability analyses. The optimal PCA-LR model retained 19 principal components, corresponding to a cumulative explained variance of 81.42%. Figure 2 presents the exploratory generalization performance of the nine classifiers.

**Figure 2:**
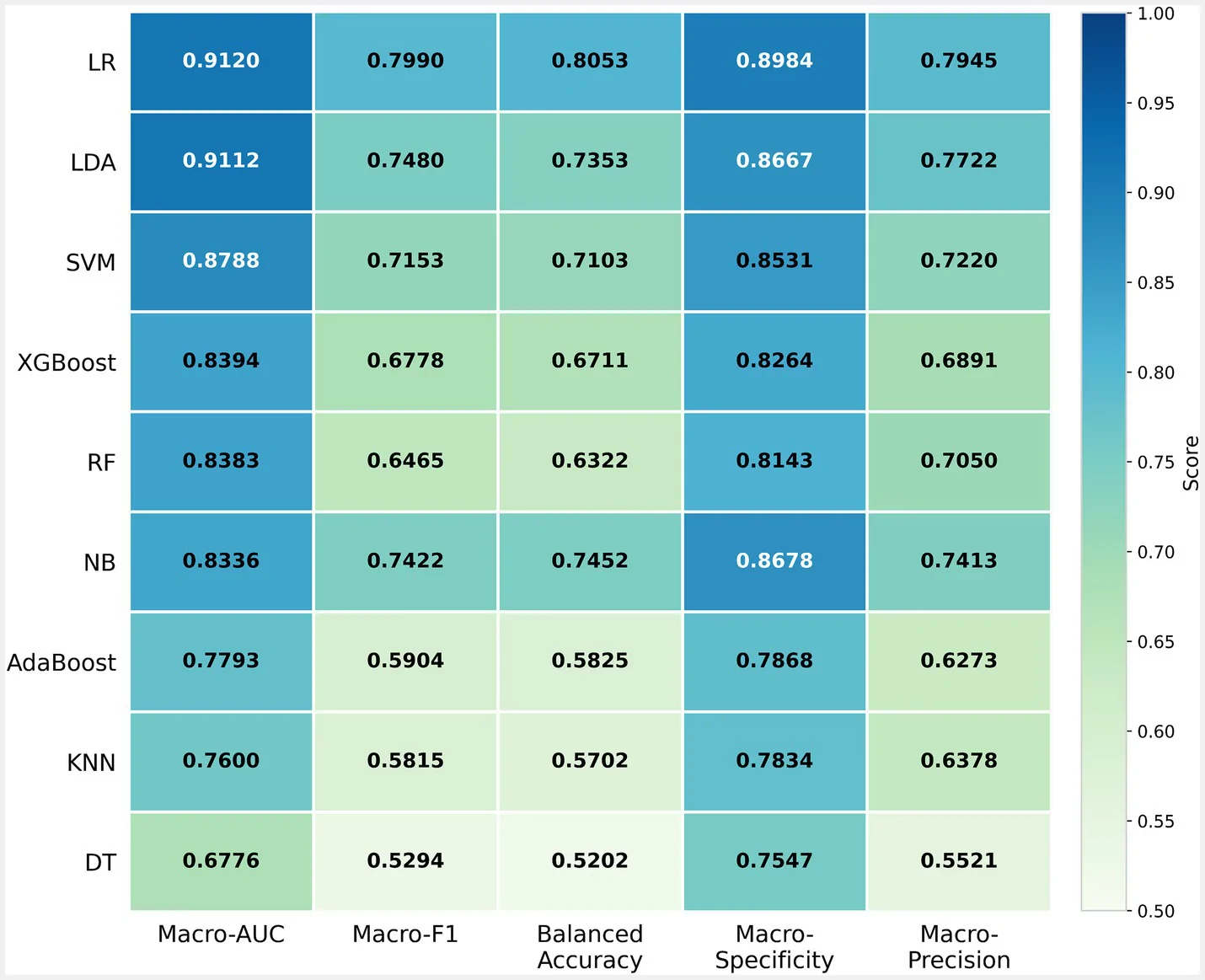
Performance matrix of 9 classifiers across five metrics. LDA, linear discriminant analysis; LR, logistic regression; SVM, support vector machine; NB, naive Bayes; RF, random forest; DT, decision tree; KNN, K-nearest neighbors; PCA, principal component analysis; ANOVA, analysis of variance; AUC, area under the curve.

Specifically, the LR model achieved a balanced accuracy of 80.5% (95% CI: 71.9–88.4%), a Macro-F1 of 0.799 (95% CI: 0.712–0.880), and a Macro-AUC of 0.912 (95% CI: 0.855–0.962), as illustrated in Figure 3A.

**Figure 3:**
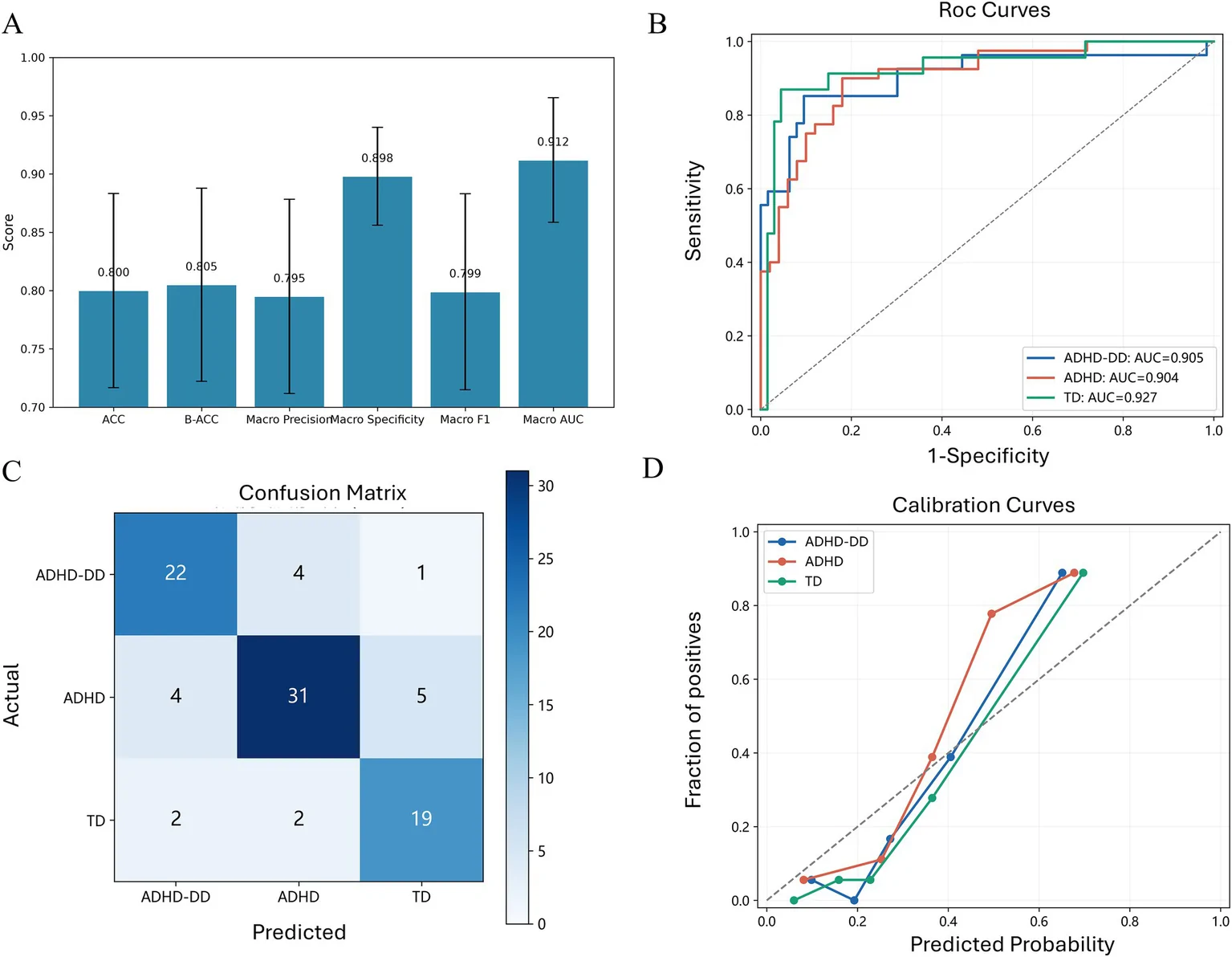
Performance evaluation of the PCA-LR model. **(A)** Overall classification metrics including accuracy (ACC), balanced accuracy (B-ACC), macro precision, macro specificity, macro F1-score, and macro-AUC. Error bars represent 95% confidence intervals. **(B)** Receiver operating characteristic (ROC) curves for OvR- multi-class classification; the dashed diagonal indicates the line of no discrimination. **(C)** Confusion matrix showing the classification results across the three groups. **(D)** Calibration curves illustrating the agreement between mean predicted probabilities and observed fractions of positives; the dashed diagonal represents perfect calibration. ACC, accuracy; B-ACC, balanced accuracy; OvR, one-vs-rest; ROC, receiver operating characteristic; AUC, area under the curve; CI, confidence interval; ADHD–DD, attention-deficit/hyperactivity disorder comorbid with developmental dyslexia; ADHD, attention-deficit/hyperactivity disorder; TD, typically developing.

### 3.4 Evaluation of the optimal model’s performance

Based on the model selection results described in Section 3.3, detailed classification performance analyses of the LR model are presented in this subsection. The LR model achieved favorable recognition performance for ADHD-DD, ADHD-only, and TD groups (Table 1). For the ADHD-DD group, the sensitivity was 0.815, specificity was 0.905, and F1-score was 0.800. For the ADHD-only group, the sensitivity was 0.775, specificity was 0.880, and F1-score was 0.805. For the TD group, the sensitivity was 0.826, specificity was 0.910, and F1-score was 0.792.

**Table 1:** Classification performance of the PCA-LR model for each diagnostic class.

Metric	ADHD–DD	ADHD	TD
Sensitivity	0.815 (0.666, 0.957)	0.775 (0.643, 0.900)	0.826 (0.647, 0.962)
Specificity	0.905 (0.828, 0.969)	0.880 (0.787, 0.962)	0.910 (0.833, 0.971)
Precision	0.786 (0.619, 0.929)	0.838 (0.714, 0.946)	0.760 (0.581, 0.917)
F1-Score	0.800 (0.667, 0.902)	0.805 (0.700, 0.892)	0.792 (0.638, 0.905)
AUC (OVR)	0.905 (0.809, 0.973)	0.904 (0.837, 0.961)	0.927 (0.838, 0.992)

Values in parentheses are 95% confidence intervals. ADHD-DD, attention-deficit/hyperactivity disorder comorbid with developmental dyslexia; ADHD, attention-deficit/hyperactivity disorder; TD, typically developing; OVR, one-vs-rest.

The ROC curves (Figure 3B) demonstrated good class-discriminating ability of the model, with AUC values of 0.905, 0.904, and 0.927 for ADHD-DD, ADHD-only, and TD, respectively. Confusion matrix analysis (Figure 3C) showed that 72 out of the 90 samples were correctly classified. Misclassifications predominantly occurred between ADHD-DD and ADHD-only subjects (four ADHD-DD samples misclassified as ADHD-only and four ADHD-only samples misclassified as ADHD-DD), indicating partial overlap between the comorbid and pure ADHD groups in the task-related EEG feature space. Fewer misclassifications were observed between the TD group and each clinical group (two cases for each pairwise comparison). The calibration curve (Figure 3D) revealed that the predicted probabilities of the optimal model generally approximated the observed frequencies across the three groups along the diagonal line, reflecting satisfactory probability calibration for all three classes. The Brier scores were 0.1241 for ADHD-DD, 0.1555 for ADHD, and 0.1024 for TD.

### 3.5 Interpretability analysis

To improve model interpretability, the contribution weights of original EEG features of the LR model were analyzed for the three classes: ADHD-DD, ADHD-only, and TD. After taking the absolute values and averaging the results across all folds, the top 10 discriminative features ranked by contribution magnitude were extracted for each class (Figure 4). These high-contribution features spanned multiple dimensions, including functional connectivity (wPLI), amplitude (Amp), graph-theoretic metrics (DC, BC, NE), nonlinear features (ApEn, SaEn, PeEn, PLZC), and time-frequency features (α-ERD/θ-ERS).

**Figure 4:**
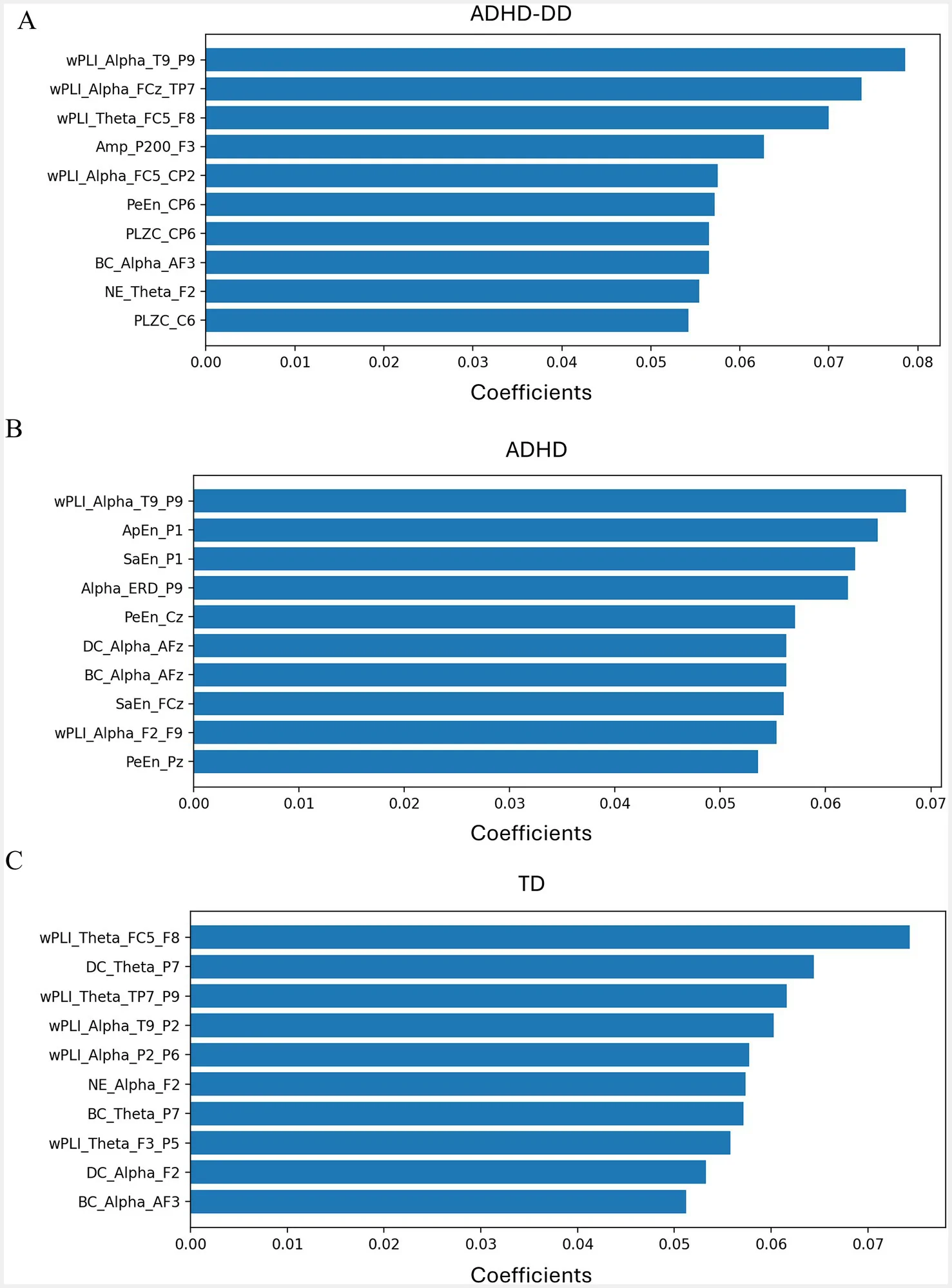
Interpretability analysis of the PCA-LR model. Bar plots illustrate the ranked contribution magnitudes of the top 10 features for the ADHD-DD comorbid group **(A)**, ADHD-only group **(B)**, and TD group **(C)**. wPLI, weighted Phase Lag Index; DC, degree centrality; NE, nodal efficiency; BC, betweenness centrality; ApEn, approximate entropy; SaEn, sample entropy; PeEn, permutation entropy; PLZC, permutation Lempel-Ziv complexity; ERD, event-related desynchronization; ADHD–DD, attention-deficit/hyperactivity disorder comorbid with developmental dyslexia; ADHD, attention-deficit/hyperactivity disorder; TD, typically developing.

The top 10 features for the ADHD-DD class included functional connectivity, ERP amplitude, nonlinear dynamics, and graph-theoretic metrics. Functional connectivity features were widely distributed across θ and α bands (e.g., wPLI_Alpha_T9_P9, wPLI_Alpha_FCz_TP7, wPLI_Alpha_FC5_CP2, wPLI_Theta_FC5_F8), with the left temporal α-band connection showing the highest weight. Other contributing features included graph-theoretic metrics (BC_Alpha_AF3, NE_Theta_F2), central-region nonlinear metrics (PeEn_CP6, PLZC_CP6, PLZC_C6), and the ERP amplitude feature (Amp_P200_F3).

For the ADHD-only class, α-band brain network metrics were prominent (wPLI_Alpha_T9_P9, DC_Alpha_AFz, BC_Alpha_AFz, wPLI_Alpha_F2_F9), with graph-theoretic metrics mainly localized at the frontal AFz electrode. Nonlinear dynamic features at parietal and central regions also showed high contribution (ApEn_P1, SaEn_P1, SaEn_FCz, PeEn_Cz, PeEn_Pz). In addition, the time-frequency feature Alpha_ERD_P9 was among the top 10 features.

Discriminative information for the TD class primarily originated from brain network features, covering both functional connectivity and graph-theoretic metrics. For functional connectivity, all selected features belonged to θ and α bands: wPLI_Theta_FC5_F8, wPLI_Theta_TP7_P9, wPLI_Alpha_T9_P2, wPLI_Alpha_P2_P6, and wPLI_Theta_F3_P5. The graph-theoretic metrics included degree centrality DC_Theta_P7 and DC_Alpha_F2, betweenness centrality BC_Theta_P7 and BC_Alpha_AF3, as well as nodal efficiency NE_Alpha_F2.

### 3.6 Model robustness validation

To evaluate the robustness of the optimal LR model, supplementary analyses were conducted from four dimensions: sex subgroup stratification, adjustment for the sex covariate, incremental value of behavioral metrics, and permutation testing. Sex subgroup analyses revealed comparable model performance between the female subgroup (B-ACC: 0.826, Macro-F1: 0.781, Macro-AUC: 0.934) and the male subgroup (B-ACC: 0.822, Macro-F1: 0.791, Macro-AUC: 0.913), both of which were similar to the primary model (B-ACC: 0.805, Macro-F1: 0.799, Macro-AUC: 0.912). Incorporating sex as a covariate did not induce substantial changes in model performance (Macro-AUC: 0.908), indicating limited confounding effects from sex. The behavior-only model yielded poor classification performance (B-ACC: 0.434, Macro-F1: 0.391, Macro-AUC: 0.684). After adding behavioral metrics to EEG features, the combined model (B-ACC: 0.774, Macro-F1: 0.772, Macro-AUC: 0.905) did not outperform the model using EEG features alone, which suggests that EEG features carried the primary predictive information. The permutation test (100 permutations) demonstrated that the true model performance exceeded all permutation-based null results (Macro-F1: 0.799, Accuracy: 0.800, Macro-AUC: 0.912; all *p* = 0.010), showing that the model performed significantly above chance level. Detailed results of these analyses are provided in Supplementary Table S8.

## 4 Discussion

This study extracted five categories of features, including ERP, time-frequency, functional connectivity, graph-theoretic and nonlinear metrics, from task-state EEG recorded during the VSWM task. Machine learning approaches were adopted to construct a three-class diagnostic model to discriminate children with ADHD-DD, ADHD-only, and TD. By systematically comparing combinations of three feature processing strategies and nine classification algorithms, PCA-LR was identified as the optimal model. This model exhibited favorable classification performance under nested cross-validation and achieved high discrimination accuracy for all three classes. Feature contribution analysis based on coefficient back-projection of the PCA-LR model further identified high-contribution features such as alpha-band functional connectivity, nonlinear features in the parietal and central regions, and θ-band brain network metrics. These findings provide feature-level clues for understanding the neurophysiological mechanisms underlying ADHD-DD.

### 4.1 Model performance and comparative analysis

Given the complexity and heterogeneity of neurodevelopmental disorder symptomatology, EEG-based machine learning approaches have been preliminarily applied to the auxiliary identification of ADHD and developmental dyslexia (DD) (Ketabi et al., 2026; Latifi et al., 2024). However, previous studies have largely focused on binary classification tasks distinguishing ADHD or DD from healthy controls (Gallego-Molina et al., 2024; Garcia-Ponsoda et al., 2024), with less attention paid to the clinical importance of comorbid populations as a distinct diagnostic entity. Moreover, most studies have relied on single-domain features, failing to integrate multidimensional information. For instance, ERP-based machine learning studies have achieved an accuracy of 69% in distinguishing children with ADHD from controls (Vahid et al., 2019). In another study, a model combining frequency domain, power spectral, and nonlinear features reached an accuracy of 86.1% (Garcia-Ponsoda et al., 2024). Distinct from these binary approaches, the present study introduces a three-class framework integrating five categories of EEG features. The overall accuracy of our model is slightly lower than that of machine learning models incorporating neuroimaging modalities such as fMRI (Alkhurayyif and Wahab Sait, 2024; Wang et al., 2026), however EEG offers advantages including cost-effectiveness, portability, and ease of acquisition. Notably, Pedrollo et al. achieved a three-class accuracy of 68.00% in distinguishing ADHD subtypes using Spiking Neural Networks (Pedrollo et al., 2022). These findings underscore the inherent complexity of multiclass classification in neurodevelopmental disorders. In this context, the 80.00% accuracy achieved in the present study reflects the effectiveness of our model in discriminating among clinically similar populations. Furthermore, the use of visuospatial working memory task-state EEG enhances the neurophysiological specificity of the extracted features by activating the shared cognitive deficit circuitry in ADHD and DD.

### 4.2 Divergent patterns of electrophysiological features revealed by interpretability analysis

To improve model transparency and clinical credibility, this study adopted a feature contribution analysis approach based on coefficient back-projection of the PCA-LR model. The classification weights in the principal component space were mapped back to the original EEG feature space to quantify the contribution of each feature to classification decisions. The results showed that model discrimination relied on differentiated combinations of multidimensional neural markers, including functional connectivity, ERP amplitudes, graph-theoretic metrics, and nonlinear dynamical entropy. Unlike many previous machine-learning studies relying on resting-state EEG, our features were derived from a VSWM task, enabling us to probe neural activity tied to cognitive processing rather than merely spontaneous baseline brain activity. These findings suggest that the discrimination of ADHD-DD, ADHD-only, and TD involves coordinated organizational strategies across distinct neural systems.

#### 4.2.1 ADHD-DD: prominent contributions of functional connectivity and early ERP components

For classification decisions of the ADHD-DD comorbid group, functional connectivity features were the most numerous and contributed most prominently, with broad temporal and spatial distributions. These connections spanned multiple brain regions including intra-frontal, intra-temporal, fronto-parietal, and centro-parietal connections within the θ (200–300 ms) and α (400–800 ms) bands. Among them, the α-band functional connectivity in the left temporal region (T9-P9) exhibited the highest weight. Chen et al. (2026) constructed three-class models for ADHD, DD and their comorbidity using resting-state EEG functional connectivity. Their results revealed a distinct discriminative pattern of functional connectivity in the comorbid group, where individual-specific functional connectivity was mainly localized in the left frontal and right parietal lobes, and this pattern significantly predicted clinical symptoms. The high contribution of multi-regional functional connectivity features in the present study echoes these findings, suggesting that widely distributed functional connectivity may serve as potential reference features for ADHD-DD classification.

Beyond inter-regional functional connectivity, features for the ADHD-DD group also included the early ERP component P200 amplitude, graph-theoretic metrics, and nonlinear dynamical features in the centro-parietal regions. The P200 component is involved in the allocation of attentional resources during the early processing of visual stimuli (Yang et al., 2025). In the context of the VSWM task, this corresponds to the encoding stage, during which children with ADHD-DD may need to recruit additional attentional resources to compensate for inefficient visuospatial encoding. Previous studies have reported enhanced P200 amplitudes in patients with ADHD or DD during neurocognitive tasks (Wu et al., 2026). Furthermore, machine learning studies have indicated that time-domain ERP metrics hold potential value for identifying isolated ADHD and dyslexia (Mohammed et al., 2026; Zaree et al., 2023). From a pathological-clinical perspective, the fronto-parietal and temporo-parietal networks participate in the early attentional encoding and integration of visuospatial information (Hnegger et al., 2011; Santangelo and Macaluso, 2013), while centro-parietal brain regions support the maintenance and updating of visuospatial working memory information (Semprini et al., 2021). The discriminative weights observed across these multi-region and multi-time-window features partly align with the clinical manifestations of visuospatial working memory deficits in children with ADHD-DD comorbidity, which involve impairments in multiple processes including attentional allocation and information maintenance.

Collectively, the discriminative information distinguishing ADHD-DD comorbidity does not rely on a single type of feature but on the combination of multidimensional information. Brain network activity, neural signal complexity, and early attention-related ERP signals within both early and late cognitive processing windows all carry individual-level discriminative value. This is consistent with the conclusions from several multimodal machine learning studies.

#### 4.2.2 ADHD: prominent contributions of nonlinear features and α-band brain networks

The high-contribution discriminative signals for the ADHD-only group originated from α-band (400–800 ms) brain network features. The α-band functional connectivity at the left temporal region (T9-P9) achieved the highest weight, and graph-theoretic metrics were predominantly localized to the midline prefrontal region. Nonlinear dynamical features from the fronto-central, central and parietal regions constituted a prominent proportion, and the α-ERD time-frequency feature at electrode P9 was also included in the high-contribution set. α-band oscillations are thought to reflect inhibitory control and the gating of task-irrelevant information, with α-ERD indicating cortical activation (Bonnefond and Jensen, 2012; Jensen and Mazaheri, 2010). Functional connectivity in the α band may facilitate efficient communication among task-relevant brain regions (Lenartowicz et al., 2016). In ADHD, atypical α-band network organization may reflect disrupted attentional modulation and inhibitory control (Ter Huurne et al., 2013). Numerous previous EEG machine learning studies have validated that α-band brain networks serve as key markers for identifying ADHD. Mao et al. (2025) adopted multidimensional EEG features combined with ensemble machine learning algorithms and identified multi-band brain network reorganization as a critical electrophysiological signature in children with ADHD. The machine learning framework reported by Mohammed et al. (2026) also identified fronto-central connectivity as a key discriminative feature. Prior studies have also demonstrated that children with ADHD exhibit significant reductions in functional connectivity and global network efficiency, alongside increased nodal efficiency within visual and dorsal attention networks (Mengjing et al., 2020). The present study further supplements relevant evidence from the perspective of individual-level discrimination.

The discriminative findings of nonlinear dynamical metrics are consistent with previous reports. Alterations in the complexity and flexibility of neural signals have been observed in individuals with ADHD (Rezaeezadeh et al., 2020; Sohn et al., 2010), and such signal differences can be captured by machine learning for individual classification. α-ERD reflects cortical activation and inhibitory control of task-relevant information (Wei and Zhou, 2020). In the VSWM task, this corresponds to the maintenance stage, during which the suppression of task-irrelevant visual information is necessary to protect the memory trace (Lenartowicz et al., 2016). Previous work has documented abnormal posterior α-ERD patterns in children with ADHD during the VSWM task, which are associated with deficits in attentional allocation and inhibitory control (Kyoung-Mi et al., 2020; Lenartowicz et al., 2016). This study identified α-ERD at the posterior parietal electrode P9 as a high-contribution feature, suggesting that ADHD discrimination may also involve altered α-band desynchronization patterns in posterior brain regions. This finding provides supplementary insights into the time-frequency contributions for ADHD classification.

#### 4.2.3 TD: discriminative contributions of brain network features

In this study, the top 10 discriminative features for the TD group were dominated by brain network metrics. Functional connectivity covered multiple interregional pathways, including intra-frontal, intra-temporal, fronto-parietal, temporo-parietal and intra-parietal connections, spanning both θ-band (200–300 ms) and α-band (400–800 ms). Among these, intra-frontal functional connectivity (FC5-F8) yielded the highest contribution. Graph-theoretic metrics were distributed across the frontal regions (F2, AF3) and temporo-parietal region (P7), without heavy concentration at any single electrode. This regional and spectral distribution aligns with the neurocognitive substrates of coordinated frontoparietal and temporoparietal network activity in TD children during visuospatial, attention, and working memory processes (Nee, 2021; Otstavnov et al., 2024; Carter and Huettel, 2013). In this classification model, the discriminative information for the TD group is not localized to a single isolated node, but is distributed across multiple regions and frequency bands. This pattern suggests that the discriminative information in TD children may reflect a global property of network-level coordination, rather than the strength of individual nodes. Most EEG machine learning studies have focused primarily on discriminating clinical groups from control subjects, with limited discussion regarding which features are critical for model classification of typically developing samples (Erolu, 2025). The present observations provide indirect support for the neural efficiency hypothesis (Neubauer and Fink, 2009). Specifically, typically developing children rely on a coordinated brain network pattern to complete visuospatial cognitive tasks, without recruiting exaggerated ERP responses or neural signal complexity outside the normal range. In addition, previous studies have reported significantly enhanced shared functional connectivity in posterior brain regions across all clinical groups, whereas such abnormalities are absent in the TD group. The discriminative pattern of the present model further corroborates that this intergroup difference can be used for individual-level classification.

#### 4.2.4 Overlap and differentiation across diagnostic groups: feature-level insights into model misclassification

Confusion-matrix analysis revealed two major sources of misclassification in the three-class model. Mutual misclassification occurred between ADHD-DD and ADHD-only groups, consistent with their substantial symptomatic overlap in clinical practice. At the feature level, the two disorder groups shared the high-weight left-temporal α-band functional connectivity (T9-P9), which ranked among the top predictors for both classes. This shared posterior network signature may partly account for their classification ambiguity. Nevertheless, clear multidimensional differentiation existed between them. High-contribution features for ADHD-DD comorbidity included broad inter-regional functional connectivity spanning both θ and α bands together with the P200 amplitude at electrode F3, whereas discriminative brain-network signals for ADHD-only were confined to the alpha band and accompanied by posterior parietal alpha-ERD. Although both groups recruited centro-parietal nonlinear dynamical metrics, their spatial distributions differed: nonlinear features clustered in posterior centro-parietal regions for ADHD-DD, whereas for ADHD-only they were distributed across fronto-central, central, and parietal electrodes.

Misclassifications also occurred between ADHD-only and TD. Although no identical top-10 discriminative features were shared between the two groups, yet high-contribution features for both groups were dominated by brain-network characteristics, including functional connectivity and graph-theoretic metrics. Despite this similarity in feature types, they diverged in spectral band distribution and spatial topography. Top-ranked brain-network features of ADHD-only were limited to the α band, while TD relied on a combination of θ- and α-band network indices distributed across frontal, temporo-parietal and parietal regions. Such patterns indicate that discrimination between ADHD-only and TD depends on group-specific network configurations rather than broad feature categories alone. This may partly account for why some ADHD-only samples were misassigned to the TD class when individual electrophysiological profiles deviated moderately from typical disease-related patterns. This combined pattern of partial overlap and multidimensional differentiation echoes clinical realities, where careful differential evaluation is required across these three child groups.

### 4.3 Limitations

This study has several limitations. First, it was a single-center study with a relatively small sample and no independent external validation set. A DD-only cohort was not included because of limited pure dyslexia participants during recruitment, which limits our ability to fully distinguish comorbidity-specific features from general dyslexia-related ones. Although nested cross-validation was used to reduce data leakage, generalizability remains to be confirmed. Second, the three groups were uneven in sample size and recruitment source (ADHD/ADHD-DD from a pediatric outpatient clinic vs. TD from the community), which may affect model stability and introduce selection bias. Third, the cross-sectional design does not allow causal inferences. Moreover, the present analyses were based primarily on EEG features, and the initial candidate features were identified through whole-sample analysis of variance (ANOVA), which may introduce a degree of selection bias. Future large-scale multicenter studies integrating multimodal data and multidimensional network analyses, as well as examining subcomponents of visuospatial working memory, are needed to further validate these findings and characterize the neuropathology of ADHD-DD comorbidity.

## 5 Conclusion

This study integrates multidimensional task-state EEG features to construct an interpretable machine learning model, providing a preliminary reference for distinguishing ADHD-DD comorbidity from ADHD and TD. Coefficient back-projection analysis suggested that the discrimination of ADHD-DD, ADHD, and TD was associated with a differentiated combination of neural oscillation patterns, and signal complexity. These preliminary feature-level patterns may offer useful clues for generating hypotheses about neural processes associated with the comorbid population, although causal interpretations and specificity to the comorbidity await validation in future four-group studies including a DD-only cohort.

## Data Availability

The raw data supporting the conclusions of this article will be made available by the authors, without undue reservation.
