# Supplementary material for: An explainable machine learning model based on multidimensional task-state electroencephalography for identifying attention-deficit/hyperactivity disorder comorbid developmental dyslexia
Source: Front Hum Neurosci. 2026 Oct 2;20:1976311. doi: 10.3389/fnhum.2026.1976311 (PMC13672606; doi:10.3389/fnhum.2026.1976311)
Supplement: Supplementary file 1 [file Table_1.docx]

**Supplementary Tables**

**Supplementary Table S1. Comparison of ERP components with significant differences among the ADHD-DD, ADHD, and TD groups.**

| **Component** | **Electrodes** | **ADHD-DD** | **ADHD** | **TD** | ***F*** | ***p*** | **FDR*_q*** |
| --- | --- | --- | --- | --- | --- | --- | --- |
| **Latency** |  |  |  |  |  |  |  |
| P100 | P3 | 112.435 (16.000) ^b^ | 124.957 (12.268) ^a^ | 119.826 (15.456) ^ab^ | 4.237 | 0.019 | 0.032 |
| **Amplitude** |  |  |  |  |  |  |  |
| N200 | P4 | -1.921 (3.657) ^b^ | -0.708 (2.520) ^ab^ | 0.157(2.834) ^a^ | 3.104 | 0.050 | 0.050 |
| P200 | F3 | 2.983 (3.072) ^a^ | 1.602 (2.008) ^ab^ | 0.698 (2.374) ^b^ | 5.555 | 0.005 | 0.019 |
| P200 | C3 | 3.281 (1.974) ^a^ | 2.256 (1.592) ^b^ | 2.212 (1.657) ^ab^ | 3.421 | 0.037 | 0.045 |
| P300 | Cz | 0.048 (1.925) ^ab^ | -0.875 (2.174) ^b^ | 0.544 (2.405) ^a^ | 3.465 | 0.036 | 0.045 |

Note: Data are mean (SD). Group differences were tested by one-way ANOVA with Bonferroni correction. ADHD-DD, attention-deficit/hyperactivity disorder comorbid with developmental dyslexia; ADHD, attention-deficit/hyperactivity disorder; TD, typical development.

**Supplementary Table S2. Time-Frequency Results of the ADHD-DD, ADHD, and TD groups.**

| **Electrodes** | **ADHD-DD** | **ADHD** | **TD** | ***F*** | ***p*** | **FDR*_q*** |
| --- | --- | --- | --- | --- | --- | --- |
| **Theta** |  |  |  |  |  |  |
| T9 | 0.011(0.115) ^ab^ | -0.042 (0.166) ^b^ | 0.059 (0.149) ^a^ | 3.521 | 0.034 | 0.044 |
| **Alpha** |  |  |  |  |  |  |
| F10 | -0.171 (0.152) ^a^ | -0.433 (0.604) ^b^ | -0.216 (0.241) ^ab^ | 3.564 | 0.033 | 0.044 |
| P5 | -0.605 (0.970) ^ab^ | -0.858 (1.330) ^b^ | -0.164 (0.286) ^a^ | 3.216 | 0.045 | 0.049 |
| P7 | -1.538 (2.077) ^ab^ | -1.839 (2.224) ^b^ | -0.597 (0.610) ^a^ | 3.192 | 0.046 | 0.049 |
| P9 | -0.859 (0.711) ^ab^ | -1.448 (1.606) ^b^ | -0.621 (0.790) ^a^ | 3.931 | 0.023 | 0.037 |

Note: Data are presented as mean (standard deviation). Group differences were assessed using one-way ANOVA with Bonferroni-corrected post-hoc comparisons. Positive values indicate event-related synchronization (ERS), whereas negative values indicate event-related desynchronization (ERD). Theta-band analysis focused on the 200–300 ms post-stimulus time window, and alpha-band analysis on the 400-800 ms post-stimulus time window. Different superscript letters (a, b, c) within the same row indicate significant group differences at *p* < 0.05. ADHD-DD, attention-deficit/hyperactivity disorder comorbid with developmental dyslexia; ADHD, attention-deficit/hyperactivity disorder; TD, typical development; ANOVA, analysis of variance. Only electrodes with significant group differences are presented. All other electrodes showed no significant group differences (*p* > 0.05).

**Supplementary Table S3. Statistical analysis of theta-band functional connectivity strength among the ADHD-DD, ADHD, and TD groups.**

| **Electrode pairs** | **ADHD-DD** | **ADHD** | **TD** | ***F*** | ***p*** | **FDR*_q*** |
| --- | --- | --- | --- | --- | --- | --- |
| **F3-P5** | 0.234 (0.125) ^a^ | 0.234 (0.122) ^a^ | 0.149 (0.072) ^b^ | 4.873 | 0.010 | 0.020 |
| **F3-P3** | 0.224 (0.118) ^a^ | 0.239 (0.129) ^a^ | 0.146 (0.072) ^b^ | 5.069 | 0.008 | 0.019 |
| **TP7-P9** | 0.263 (0.116) ^a^ | 0.248 (0.141) ^a^ | 0.161 (0.091) ^b^ | 5.041 | 0.008 | 0.019 |
| **FC5-F8** | 0.235 (0.109) ^a^ | 0.193 (0.091) ^ab^ | 0.150 (0.077) ^b^ | 5.149 | 0.008 | 0.019 |

Note: Data are presented as mean (standard deviation). Group differences were assessed using one-way ANOVA with Bonferroni-corrected post-hoc comparisons. Theta-band functional connectivity analysis focused on the 200-300 ms post-stimulus time window. Different superscript letters (a, b, c) within the same row indicate significant group differences at *p* < 0.05. ADHD-DD, attention-deficit/hyperactivity disorder comorbid with developmental dyslexia; ADHD, attention-deficit/hyperactivity disorder; TD, typical development; ANOVA, analysis of variance. **Supplementary Table S4. Statistical analysis of alpha-band functional connectivity strength among the ADHD-DD, ADHD, and TD groups.**

| **Electrode pairs** | **ADHD-DD** | **ADHD** | **TD** | ***F*** | ***p*** | **FDR*_q*** |
| --- | --- | --- | --- | --- | --- | --- |
| **F2-F9** | 0.179 (0.083) ^a^ | 0.125 (0.041) ^b^ | 0.168 (0.089) ^a^ | 5.788 | 0.004 | 0.019 |
| **FCz-TP7** | 0.192 (0.093) ^a^ | 0.135 (0.054) ^b^ | 0.142 (0.060) ^b^ | 5.879 | 0.004 | 0.019 |
| **T9-P9** | 0.190 (0.091) ^a^ | 0.136 (0.046) ^b^ | 0.139 (0.074) ^b^ | 5.412 | 0.006 | 0.019 |
| **F2-O1** | 0.175 (0.079) ^b^ | 0.168 (0.068) ^b^ | 0.230 (0.094) ^a^ | 4.869 | 0.010 | 0.020 |
| **T9-P2** | 0.192 (0.069) ^ab^ | 0.161 (0.069) ^a^ | 0.135 (0.032) ^b^ | 5.518 | 0.006 | 0.019 |
| **FC5-CP2** | 0.196 (0.080) ^a^ | 0.146 (0.054) ^b^ | 0.149 (0.058) ^b^ | 5.428 | 0.006 | 0.019 |
| **C1-CP2** | 0.198 (0.072) ^a^ | 0.154 (0.065) ^b^ | 0.148 (0.049) ^b^ | 5.198 | 0.007 | 0.019 |
| **FT7-CP2** | 0.191 (0.070) ^a^ | 0.148 (0.056) ^b^ | 0.133 (0.048) ^b^ | 6.877 | 0.002 | 0.019 |
| **P2-P6** | 0.201 (0.093) ^a^ | 0.187 (0.077) ^a^ | 0.132 (0.047) ^b^ | 5.621 | 0.005 | 0.019 |
| **AF4-FC4** | 0.248 (0.124) ^a^ | 0.174 (0.075) ^b^ | 0.276 (0.161) ^a^ | 6.372 | 0.003 | 0.019 |
| **F2-FC4** | 0.250 (0.144) ^ab^ | 0.195 (0.083) ^b^ | 0.316 (0.174) ^a^ | 6.289 | 0.003 | 0.019 |
| **Fz-FC4** | 0.223 (0.135) ^ab^ | 0.168 (0.070) ^b^ | 0.265 (0.133) ^a^ | 5.986 | 0.004 | 0.019 |
| **AF4-FC6** | 0.206 (0.107) ^ab^ | 0.149 (0.065) ^b^ | 0.248 (0.155) ^a^ | 6.738 | 0.002 | 0.019 |
| **F2-FC6** | 0.200 (0.093) ^ab^ | 0.167 (0.065) ^b^ | 0.261 (0.175) ^a^ | 5.327 | 0.007 | 0.019 |
| **FC4-F4** | 0.254 (0.137) ^ab^ | 0.188 (0.077) ^b^ | 0.289 (0.183) ^a^ | 4.987 | 0.009 | 0.020 |
| **FC6-F4** | 0.217 (0.112) ^ab^ | 0.165 (0.063) ^b^ | 0.266 (0.192) ^a^ | 5.154 | 0.008 | 0.019 |

Note: Data are presented as mean (standard deviation). Group differences were assessed using one-way ANOVA with Bonferroni-corrected post-hoc comparisons. Alpha-band functional connectivity analysis focused on the 400-800 ms post-stimulus time window. Different superscript letters (a, b, c) within the same row indicate significant group differences at *p* < 0.05. ADHD-DD, attention-deficit/hyperactivity disorder comorbid with developmental dyslexia; ADHD, attention-deficit/hyperactivity disorder; TD, typical development; ANOVA, analysis of variance.

**Supplementary Table S5. Comparison of theta-band graph-theoretical metrics among the ADHD-DD, ADHD, and TD groups.**

| **Electrodes** | **ADHD-DD** | **ADHD** | **TD** | ***F*** | ***p*** | **FDR*_q*** |
| --- | --- | --- | --- | --- | --- | --- |
| **NE** |  |  |  |  |  |  |
| F2 | 0.165 (0.035) ^b^ | 0.175 (0.023) ^ab^ | 0.185 (0.028) ^a^ | 3.166 | 0.047 | 0.049 |
| F9 | 0.162 (0.051) ^b^ | 0.181 (0.037) ^ab^ | 0.189 (0.021) ^a^ | 3.234 | 0.044 | 0.049 |
| P7 | 0.197 (0.037) ^a^ | 0.190 (0.035) ^ab^ | 0.167 (0.054) ^b^ | 3.564 | 0.033 | 0.044 |
| **BC** |  |  |  |  |  |  |
| P7 | 13.290 (13.493) ^a^ | 9.615 (8.721) ^ab^ | 3.764 (4.019) ^b^ | 5.944 | 0.004 | 0.019 |
| P9 | 32.283 (36.465) ^a^ | 16.362 (19.712) ^b^ | 16.762 (20.349) ^ab^ | 3.357 | 0.039 | 0.047 |
| Cz | 5.232 (9.471) ^ab^ | 7.656 (11.846) ^b^ | 14.990 (19.414) ^a^ | 3.327 | 0.041 | 0.048 |
| **DC** |  |  |  |  |  |  |
| P7 | 6.428 (2.926) ^a^ | 5.915 (2.808) ^a^ | 4.129 (2.515) ^b^ | 4.541 | 0.013 | 0.025 |
| CP2 | 4.169 (3.051) ^ab^ | 4.699 (3.482) ^a^ | 2.542 (2.449) ^b^ | 3.443 | 0.036 | 0.045 |

Note: Data are presented as mean (standard deviation). Group differences were assessed using one-way ANOVA with Bonferroni-corrected post-hoc comparisons. Theta-band graph-theoretical metrics were calculated based on functional connectivity in the 200-300 ms post-stimulus time window. Different superscript letters (a, b, c) within the same row indicate significant group differences at *p* < 0.05. ADHD-DD, attention-deficit/hyperactivity disorder comorbid with developmental dyslexia; ADHD, attention-deficit/hyperactivity disorder; TD, typical development; NE, nodal efficiency; BC, betweenness centrality; DC, degree centrality.

**Supplementary Table S6. Comparison of alpha-band graph-theoretical metrics among the ADHD-DD, ADHD, and TD groups.**

| **Electrodes** | **ADHD-DD** | **ADHD** | **TD** | ***F*** | ***p*** | **FDR*-q*** |
| --- | --- | --- | --- | --- | --- | --- |
| **NE** |  |  |  |  |  |  |
| AF4 | 0.180 (0.027) ^ab^ | 0.170 (0.037) ^b^ | 0.196 (0.017) ^a^ | 5.603 | 0.005 | 0.019 |
| F2 | 0.176 (0.039) ^b^ | 0.179 (0.029) ^b^ | 0.199 (0.017) ^a^ | 4.256 | 0.017 | 0.031 |
| **BC** |  |  |  |  |  |  |
| F2 | 4.988 (4.613) ^b^ | 6.464 (8.55) ^b^ | 11.820 (10.382) ^a^ | 4.668 | 0.012 | 0.024 |
| F4 | 4.740 (4.747) ^b^ | 5.237 (4.513) ^b^ | 9.780 (11.291) ^a^ | 3.888 | 0.024 | 0.037 |
| Fz | 6.307 (7.179) ^ab^ | 5.140 (4.591) ^b^ | 9.680 (8.183) ^a^ | 3.520 | 0.034 | 0.044 |
| AF3 | 3.046 (3.225) ^b^ | 4.765 (5.090) ^ab^ | 7.875 (7.457) ^a^ | 4.949 | 0.009 | 0.020 |
| AF4 | 4.860 (4.708) ^b^ | 4.139 (5.327) ^b^ | 9.748 (7.894) ^a^ | 6.769 | 0.002 | 0.019 |
| AFz | 8.956 (9.905) | 4.670 (4.350) | 8.692 (7.219) | 3.650 | 0.030 | 0.044 |
| **DC** |  |  |  |  |  |  |
| F2 | 4.371 (2.058) ^b^ | 4.341 (1.802) ^b^ | 5.793 (1.823) ^a^ | 4.815 | 0.010 | 0.020 |
| AF4 | 4.420 (2.322) ^ab^ | 3.940 (1.952) ^b^ | 5.521 (1.652) ^a^ | 4.423 | 0.015 | 0.028 |
| AFz | 5.185 (2.281) | 4.208 (1.584) | 5.363 (1.708) | 3.573 | 0.032 | 0.044 |

Note: Data are presented as mean (standard deviation). Group differences were assessed using one-way ANOVA with Bonferroni-corrected post-hoc comparisons. Alpha-band graph-theoretical metrics were calculated based on functional connectivity in the 400-800 ms post-stimulus time window. Different superscript letters (a, b, c) within the same row indicate significant group differences at *p* < 0.05. Rows without superscript letters indicate significant ANOVA results (*p* < 0.05) but non-significant post-hoc pairwise comparisons. Abbreviations: ADHD-DD, attention-deficit/hyperactivity disorder comorbid with developmental dyslexia; ADHD, attention-deficit/hyperactivity disorder; TD, typical development; NE, nodal efficiency; BC, betweenness centrality; DC, degree centrality.

**Supplementary Table S7. Comparison of nonlinear dynamics measures among the ADHD-DD, ADHD, and TD groups.**

| **Electrodes** | **ADHD-DD** | **ADHD** | **TD** | ***F*** | ***p*** | **FDR*-q*** |
| --- | --- | --- | --- | --- | --- | --- |
| **ApEn** |  |  |  |  |  |  |
| P1 | 0.005 (0.009) ^a^ | -0.001 (0.009) ^b^ | 0.003 (0.009) ^ab^ | 4.187 | 0.018 | 0.032 |
| **SaEn** |  |  |  |  |  |  |
| FCz | 0.001 (0.008) ^ab^ | -0.003 (0.010) ^b^ | 0.004 (0.009) ^a^ | 3.814 | 0.026 | 0.039 |
| P1 | 0.006 (0.010) | 0.000 (0.010) | 0.005 (0.010) | 3.264 | 0.043 | 0.049 |
| **PeEn** |  |  |  |  |  |  |
| C3 | 0.001 (0.004) ^ab^ | 0.002 (0.004) ^a^ | -0.001 (0.006) ^b^ | 4.052 | 0.021 | 0.034 |
| C5 | 0.001 (0.004) ^a^ | 0.001 (0.004) ^a^ | -0.002 (0.005) ^b^ | 5.247 | 0.007 | 0.019 |
| TP7 | 0.001 (0.004) ^a^ | 0.001 (0.003) ^ab^ | -0.001 (0.003) ^b^ | 3.802 | 0.026 | 0.039 |
| Pz | 0.006 (0.005) ^ab^ | 0.004 (0.005) ^b^ | 0.008 (0.005) ^a^ | 5.103 | 0.008 | 0.019 |
| CP6 | 0.004 (0.004) ^a^ | 0.001 (0.004) ^b^ | 0.000 (0.004) ^b^ | 6.282 | 0.003 | 0.019 |
| TP8 | 0.002 (0.003) ^a^ | 0.001 (0.003) ^ab^ | 0.000 (0.003) ^b^ | 3.204 | 0.045 | 0.049 |
| C6 | 0.002 (0.003) ^a^ | 0.000 (0.003) ^ab^ | -0.001 (0.004) ^b^ | 6.461 | 0.002 | 0.019 |
| PO3 | 0.008 (0.006) | 0.005 (0.005) | 0.008 (0.005) | 3.538 | 0.033 | 0.044 |
| Cz | 0.002 (0.004) | 0.001 (0.004) | 0.003 (0.003) | 3.108 | 0.050 | 0.050 |
| **PLZC** |  |  |  |  |  |  |
| C3 | 0.001 (0.003) ^ab^ | 0.001 (0.003) ^a^ | -0.001 (0.005) ^b^ | 3.193 | 0.046 | 0.049 |
| C5 | 0.001 (0.003) ^a^ | 0.001 (0.003) ^a^ | -0.002 (0.003) ^b^ | 5.805 | 0.004 | 0.019 |
| Pz | 0.005 (0.004) ^ab^ | 0.003 (0.003) ^b^ | 0.005 (0.004) ^a^ | 4.159 | 0.019 | 0.032 |
| CP6 | 0.003 (0.003) ^ab^ | 0.001 (0.003) ^b^ | 0.000 (0.003) ^b^ | 5.110 | 0.008 | 0.019 |
| C6 | 0.002 (0.002) ^a^ | 0.000 (0.002) ^ab^ | -0.001 (0.003) ^b^ | 5.919 | 0.004 | 0.019 |
| F2 | 0.001 (0.002) | 0.002 (0.003) | 0.001 (0.002) | 3.104 | 0.050 | 0.050 |

Note: Data are presented as mean (standard deviation). Group differences were assessed using one-way ANOVA with Bonferroni-corrected post-hoc comparisons. Different superscript letters (a, b, c) within the same row indicate significant group differences at *p* < 0.05. Rows without superscript letters indicate significant ANOVA results (*p* < 0.05) but non-significant post-hoc pairwise comparisons. ADHD-DD, attention-deficit/hyperactivity disorder comorbid with developmental dyslexia; ADHD, attention-deficit/hyperactivity disorder; TD, typical development; ApEn, approximate entropy; SaEn, sample entropy; PeEn, permutation entropy; PLZC, permutation Lempel-Ziv complexity; ANOVA, analysis of variance.

**Supplementary Table S8. Sensitivity analyses of the optimal PCA-LR model.**

| **Model** | **ACC** | **B-ACC** | **Macro-AUC** | **Macro-F1** |
| --- | --- | --- | --- | --- |
| LR (EEG + Behavior) | 0.773 | 0.774 | 0.905 | 0.772 |
| LR (Behavior only) | 0.386 | 0.434 | 0.684 | 0.391 |
| LR (Main model, female) | 0.800 | 0.826 | 0.934 | 0.781 |
| LR (Main model, male) | 0.800 | 0.822 | 0.913 | 0.791 |
| LR (EEG + Sex as covariate) | 0.800 | 0.801 | 0.908 | 0.799 |
